# Inflammatory myofibroblastic tumor of the heart in an older woman with paroxysmal atrial fibrillation: a case report and review of the literature

**DOI:** 10.1186/s13019-024-02525-0

**Published:** 2024-02-03

**Authors:** Fu-Rong Luo, Yi-Fen Lin, Jing-Lian Lin, Xiao-Shan Liang, Hui-Jun Xiao, Rui-Gang Huang

**Affiliations:** Department of Radiology, Zhangzhou Affiliated Hospital of Fujian Medical University, Zhangzhou, China

**Keywords:** Inflammatory myofibroblastic tumor, Heart, CT, MRI

## Abstract

Inflammatory myofibroblastic tumors (IMTs) of the heart are rarely observed in the eldly. We report a case involving an elderly woman with an IMT situated on the right atrial wall. The tumor was fully excised. The patient had a smooth recovery post-surgery and remained free of recurrence for three years.

## Introduction

Cardiac inflammatory myofibroblastic tumors (IMTs) are exceptionally rare, representing less than 5% of all primary cardiac tumors [1]. While these tumors can present at any age, they are predominantly observed in children. In literature scarcely ten cases have been recorded involving individuals aged 60 or older. Surgical excision remains the recommended treatment strategy for these tumors. We describe a case of an IMT in an elder woman diagnosed with paroxysmal atrial fibrillation. The tumor was not only effectively excised, but the patient also underwent a concurrent atrial fibrillation ablation. Her post-operative recovery was commendable.

## Case description

**Fig. 1:**
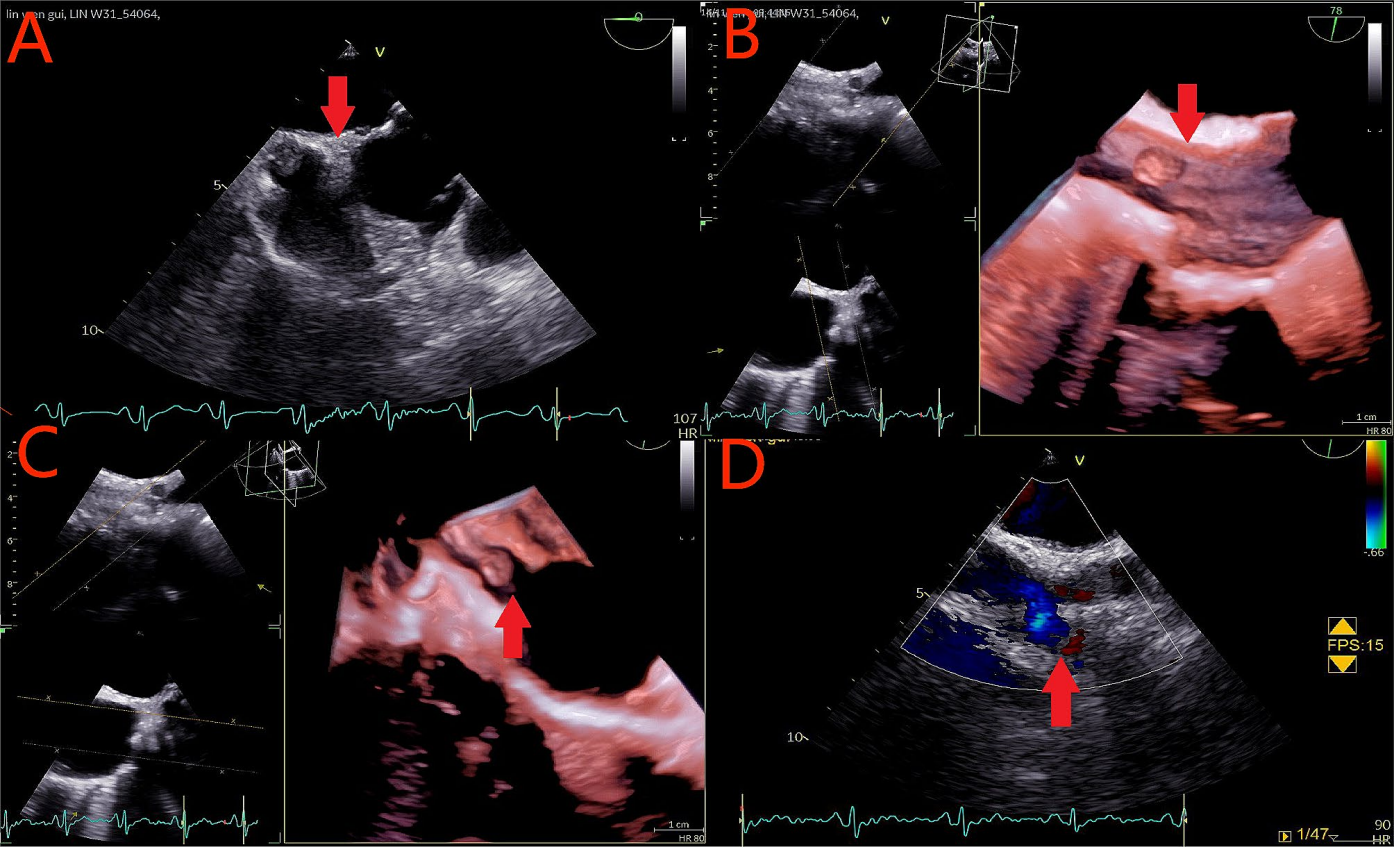
**A** ~ **C**: A solid hypoechoic nodule of 11 mm×10 mm in size on the right atrial surface of the atrial septum near the entrance of the superior vena cava, with uniform internal echo and a base width of about 5 mm. **D**: CDFI: there was no obvious blood flow signal in the nodule. Mass indicated in Figures a-d by arrows

**Fig. 2:**
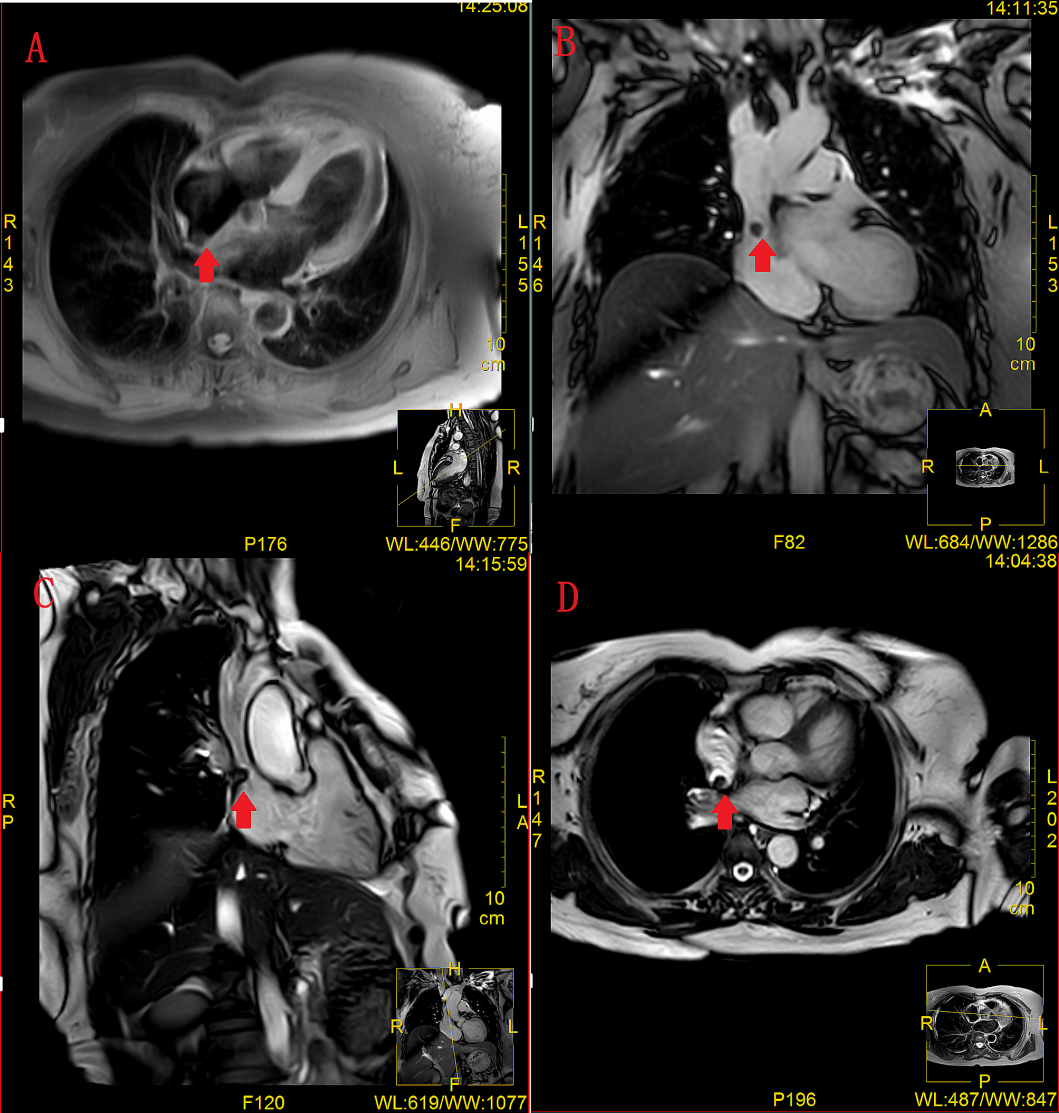
Nodular shadow near the entrance of superior vena cava of the right atrium, isot one nodule, well-defined, uniform signal, size 10 mm × 9 mm, wide base, close to the right atrial septum, about 5.4 mm. Mass indicated in Figures a-d by arrows

A 62-year-old woman came to us, reporting palpitations and dizziness over the past five months without any clear onset triggers. Each episode lasted from 10 s to an hour and resolved independently. She had previously been treated at a local hospital and diagnosed as: (1) Arrhythmia: rapid atrial fibrillation; (2) Hypertension. Following treatment, she was discharged, but her symptoms persisted. Currently, the patient has sought care at our hospital for further examination and treatment. Upon admission, her physical examination revealed a blood pressure of 136/82mmHg, a marginally enlarged heart boundary, and an auscultatory heart rate of 72 beats/min with arrhythmia. Laboratory findings including routine blood, ten items of urine + sediment, fecal occult blood + stool routine, liver function, renal function, electrolytes, blood lipids, four coagulation tests, plasma d-dimer test, B-type urinary natriuretic peptide, ultrasensitive C-reactive protein, thyroid function test, and lipoprotein were all within normal ranges. Coronary artery and left atrium angiography results were normal. Transesophageal echocardiography showed a solid neoplasm on the right atrial surface of the atrial septum with a small amount of tricuspid regurgitation (Fig. 1). Cardiac magnetic resonance imaging (MRI) indicated the mass, adjacent to the entrance of the superior vena cava was situated in the right atrium and near the entrance to the superior vena cava (displaying isointensity on T1WI sequence, a well-defined, uniform signal, measuring 10 mm x 9 mm, with a base width of approximately 5.4 mm, and proximate to the right atrial septum). The cardiac dynamic film showed that the mass, having a broad base, was anchored near the entrance of the superior vena cava of the right atrium, but without causing significant obstruction at its entry during cardiac contractions. (Fig. 2).

**Fig. 3:**
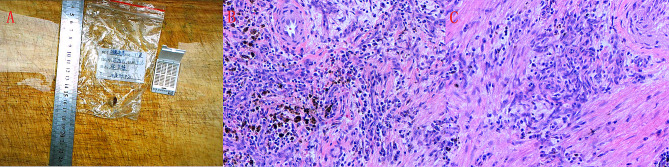
**A**: Gross pathology specimen, **B&C**: Microscopic view. Microscopically, many fibroblasts were arranged in bundles, slight vessel hyperplasia, interstitial myxoid change or collagenization, infiltration of lymphocytes and plasma cells in the background, focal hemorrhage, and hemosiderosis

Prior to the procedure, a multidisciplinary team comprising specialists from cardiology, cardiac surgery, radiology, and oncology collaborated to finalize the preoperative diagnosis and surgical plan. The patient then underwent thoracoscopic removal of the right atrial tumor and atrial fibrillation radiofrequency ablation under general anesthesia, using cardiopulmonary bypass and cardiac arrest. During the procedure, we observed a mildly enlarged heart. The left atrium was thrombosis-free. Upon opening the right atrium, a solid cardiac tumor was found on the right side of the interatrial septum, measuring approximately 10 mm x 10 mm. The tumor was precisely excised along its base, with the remnants cauterized, ensuring a safe distance from the conduction bundle. The endocardium was then sutured to restore continuity. The left atrial wall was folded along the left auricle, and ablation was performed on the atrial wall following the maze pattern used for atrial fibrillation surgical procedures. Postoperative pathology indicated IMT: SMA (+), Desmin (partially positive), Vimentin (+), S-100 (-), CD68 (-), Bcl-2 (+), CD34 (vascular positive), ALK(D5F3) (-), H-Caldesmon (partially positive), and CK-P (-) (Fig. 3). The tumor showed no local or distant invasions, and further chemotherapy was not administered. Fourteen days post-operation, the patient was discharged smoothly. Subsequent ECG showed a return to normal sinus rhythm, with partial ST-T changes but no atrial fibrillation. Regular outpatient follow-ups with appropriate medication were conducted post-discharge, followed by biannual check-ups primarily involving physical examination, ECG, and organ ultrasonography. Over the course of a 3-year follow-up, the patient remained recurrence-free.

## Discussion

IMT represents a unique and rare mesenchymal tumor. It is characterized by the World Health Organization (WHO) as a mesenchymal neoplasm comprised of differentiated myofibroblasts and spindle cells, frequently accompanied by an abundance of plasma cells and lymphocytes. Additionally, variations in myxedema and the presence of small blood vessels can be observed. Historically, IMT has been referred to by various names such as inflammatory pseudotumor, plasma cell granuloma, fibroxanthoma, inflammatory myofibroblastic hyperplasia, and myxoid hamartoma. The label “Inflammatory pseudotumor (IPT)” gained popularity due to the pronounced presence of inflammatory cells within the tumor. However, understanding the true nature of IMT’s origin has led to its differentiation from IPT, making it evident that they are two distinct entities [2]. A notable finding is the association of IMT with gene rearrangements of the anaplastic lymphoma kinase, underscoring IMT’s tumorigenic nature. Within the IPT classification, certain studies have highlighted the significance of IgG4-positive plasma cells in its pathogenesis [3]. Zhu and colleagues established criteria for distinguishing between IMT and IgG4-related IPT. Clear distinctions exist in the clinical and pathological features of IMT versus IgG4-related IPT, with cytologic atypia, IgG4, and Alk-1 staining playing pivotal roles in their differential diagnosis [2]. 

The etiology of cardiac IMT remains unclear. Potential causes include immune responses to infectious agents such as HHV-8 and EBV, reactions to injury [4], and chromosomal aberrations, specifically in region 23 of the short arm of chromosome 2 [5]. While IMT most commonly manifests in the lungs, it can also appear in various other organs and tissues, including the liver, spleen, bladder, lymph nodes, and paranasal sinuses.

IMT is predominantly observed in children and young adults [6]. The differences between IMT in children and adults were showed as follow: (1) Incidence: IMT in children is relatively more commonly reported. (2) Sites of occurrence: In children, IMT primarily occurs in the lungs, while in adults, IMT can appear in various parts of the body, including the lungs, retroperitoneum, pelvis, and intestines. (3) Genetic features: Some IMTs exhibit ALK (anaplastic lymphoma kinase) gene rearrangement. This rearrangement occurs at a higher rate in pediatric IMTs compared to adult IMTs. (4) Clinical manifestations: Although symptoms of IMT in children and adults might be similar, the clinical symptoms may vary due to different locations. For instance, a pulmonary IMT might lead to symptoms like coughing, breathing difficulties, or chest pain, while an abdominal IMT might result in abdominal pain or indigestion. (5) Malignant potential: While most IMTs are considered benign, some might relapse or metastasize. This malignant potential might be slightly higher in adults. (6) Treatment: The choice of treatment for IMT depends on the tumor’s size, location, and the presence of ALK rearrangement. Surgical resection is the primary treatment choice. If the tumor shows ALK rearrangement, ALK inhibitors, such as Crizotinib, might be considered as a treatment option.

Cardiac IMT is uncommon, often captured in isolated case reports. Among these cases, it is more prevalent in females, with a higher incidence in children. There are fewer reports in the English literature about individuals over the age of 60. While IMT can manifest anywhere in the heart, it’s frequently reported in the right ventricular outflow tract, right atrium, and left ventricle. Depending on the tumor’s location and size, clinical manifestations can vary widely, ranging from asymptomatic to sudden death. Some cases might present with an inflammatory-related clinical course [7]. In this case, an elderly woman was incidentally diagnosed with a cardiac tumor during a check-up prompted by paroxysmal atrial fibrillation. The tumor was relatively small and showed no significant obstructive symptoms. Notably, this represents the only documented case in English literature where an elderly patient with a cardiac tumor also exhibited paroxysmal atrial fibrillation. Cardiac IMT may contribute to atrial fibrillation [8]. Histologically, inflammatory myofibroblastic tumors show features like mucoid collagenization, chronic inflammatory cells, and calcification, resembling changes in myocardial infarction. This may also be a cause of arrhythmia. Additionally, some cardiac tumors can cause atrial fibrillation by altering the heart’s structure and function or by compressing or damaging surrounding tissues. However, not all atrial fibrillation cases stem from tumors. More prevalent triggers encompass hypertension, coronary ailments, heart valve diseases, heart failure, and diabetes [9]. There is no strong evidence or examination proving a correlation between IMT and atrial fibrillation. Considering patient’s advanced age and hypertension history, she was already at a heightened risk for atrial fibrillation. A thorough pre-operative assessment, routinely completed with ECG, cardiac ultrasound, and imaging techniques such as CTA/MRA, is essential for accurate diagnosis [6]. 

Three-dimensional transesophageal echocardiography (3D-TEE) is a diagnostic modality for cardiac tumors, assessing tumor dimensions across various planes. Both computed tomography (CT) and magnetic resonance imaging (MRI) are vital diagnostic tools for cardiac tumors. MRI can histologically characterize some cardiac tumors using sequences like T1-weighted (T1WI), T2-weighted (T2WI), and diffusion-weighted imaging (DWI) in combination with contrast enhancement [10]. The literature provides limited descriptions of the imaging features of cardiac inflammatory myofibroblastic tumors (IMT). Hoey ET detailed a cardiac IMT case showcasing lobulated contours, broad basal attachment, myocardial infiltration, and delayed enhancement [11]. D’Angelo described another cardiac IMT which was hyperintense on T2WI sequences, heterogeneously isointense on T1WI sequences, distinguishable from thrombi, and had early or late gadolinium enhancement differentiating it from myxomas and low-grade sarcomas [10]. In the presented case, both 3D-TEE and MRI were utilized, effectively capturing the lesion’s location, broad base, and myocardial attachment. MRI revealed a heterogeneously and mildly hypointense signal on the T2-WI sequence. On cine images, the tumor exhibited synchronous pulsation with cardiac motion. Given the neoplasm’s diminutive size at diagnosis, MRI contrast enhancement was not pursued, resulting in atypical imaging characteristics. In summary, due to the infrequent occurrence of cardiac IMT, MRI features largely depend on variations in tissue composition, mucoid content, and cellular proportion.

A definitive diagnosis of IMT requires confirmation through postoperative histopathology. Predominantly, myofibroblasts are arranged in bundles, with the histological pattern characterized by increased inflammatory infiltrates. This case tested negative for anaplastic lymphoma kinase (ALK) (D5F3). However, a limitation was that fluorescence in situ hybridization was not conducted. IMT is typically considered to exhibit indeterminate behavior, often being locally aggressive but seldom metastasizing distantly [2]. Some literature alludes to recurrence, but a comprehensive study on recurrence rates remains to be done [12]. Surgical intervention is favored for cardiac IMT, due to the looming risk of complications like prolapse or embolism. Careful dissection of the tumor base and the avoidance of conduction tissues and valves are imperative. In this instance, the tumor’s volume was minimal, and it was entirely excised during the procedure. Given the acknowledged risk of recurrence, vigilant medical surveillance, encompassing consistent postoperative echocardiography, is crucial. Moreover, there have been citations concerning the employment of steroids in treating cardiac IMT. Observations indicated a reduction and eventual subsidence in tumor size, followed by a recurrence in some cases [13, 14]. A few scholars contend that steroid treatments may spur proliferation, contributing to tumor enlargement [15]. 

There have been several reports on the use of chemotherapy in IMT patients, predominantly as neoadjuvant therapy to reduce tumor size before surgical resection. Cytotoxic chemotherapy, especially those based on anthracycline and methotrexate with or without Vinorelbine/vinblastine regimens, has been suggested as a treatment option [16]. In recent times, the association between ALK and IMT has been acknowledged. While ALK expression is characteristic of IMT, it isn’t a definitive marker. Roughly half of all cases exhibit ALK gene rearrangements. These rearrangements are more prevalent in pediatric IMT compared to adult IMT where their occurrence is notably lower [17]. Additionally, fusions involving Ros1, PDGFR β, Ret, and NTRK have been documented. There’s a growing interest in ALK inhibitors and targeted therapies as alternatives to surgery and chemotherapy. Specifically, crizotinib, which targets IMT with a positive ALK gene mutation, has been shown to induce tumor shrinkage and enhance the outcomes of surgical interventions. It appears to be an optimal treatment choice for IMT cases that are challenging to resect or prone to recurrence [18, 19]. 

## Conclusion

In this case, the cardiac IMT was successfully resected, and the short-term results have been stable. Further monitoring is needed for the long-term effects.

## Data Availability

The data supporting this study’s findings are available on request from the corresponding author. The data are not available to the public due to privacy or ethical restrictions.

## References

[1] Mizia-Malarz A, Sobol-Milejska G, Buchwald J, Woś H (2016). Inflammatory myofibroblastic tumor of the heart in the infant: review of the literature. J Pediatr Hematol Oncol.

[2] Zhu L, Li J, Liu C, Ding W, Lin F, Guo C, Liu L (2017). Pulmonary inflammatory myofibroblastic tumor versus IgG4-related inflammatory pseudotumor: differential diagnosis based on a case series. J Thorac Dis.

[3] Zen Y, Kitagawa S, Minato H, Kurumaya H, Katayanagi K, Masuda S, Niwa H, Fujimura M, Nakanuma Y (2005). IgG4-positive plasma cells in inflammatory pseudotumor (plasma cell granuloma) of the lung. Hum Pathol.

[4] Mergan F, Jaubert F, Sauvat F, Hartmann O, Lortat-Jacob S, Révillon Y, Nihoul-Fékété C, Sarnacki S (2005). Inflammatory myofibroblastic tumor in children: clinical review with anaplastic lymphoma kinase, Epstein-Barr virus, and human herpesvirus 8 detection analysis. J Pediatr Surg.

[5] Ma Z, Hill DA, Collins MH, Morris SW, Sumegi J, Zhou M, Zuppan C, Bridge JA (2003). Fusion of ALK to the ran-binding protein 2 (RANBP2) gene in inflammatory myofibroblastic tumor. Genes Chromosomes Cancer.

[6] Eilers AL, Nazarullah AN, Shipper ES, Jagirdar JS, Calhoon JH, Husain SA (2014). Cardiac inflammatory myofibroblastic tumor: a comprehensive review of the literature. World J Pediatr Congenit Heart Surg.

[7] Kato T, Tomita S, Tamaki M, Yutani C, Okawa Y (2014). Inflammatory myofibroblastic tumor of the heart. Heart Vessels.

[8] Barriales Alvarez V, Morís de la Tassa C, Sánchez Posada I, Barriales Villa R, Rubin López J, de la Hera Galarza JM, Vara Manso J, Hevia Nava S (1999). Cortina Llosa A. [The etiology and associated risk factors in a sample of 300 patients with atrial fibrillation]. Rev Esp Cardiol.

[9] Falk RH (1998). Etiology and complications of atrial fibrillation: insights from pathology studies. Am J Cardiol.

[10] D’Angelo T, Mazziotti S, Inserra MC, De Luca F, Agati S, Magliolo E, Pathan F, Blandino A, Romeo P (2019). Cardiac inflammatory myofibroblastic tumor. Circ Cardiovasc Imaging.

[11] Hoey ET, Ganesh V, Gopalan D, Screaton NJ (2009). Cardiac inflammatory myofibroblastic tumor: evaluation with dual-source CT. J Cardiovasc Comput Tomogr.

[12] 12, Domínguez-Massa C, Doñate-Bertolín L, Blanco-Herrera ÓR, Heredia-Cambra T, Pérez-Guillén M, Martínez-Cózar V, Mayordomo-Aranda E, Hornero-Sos F. Inflammatory myofibroblastic tumor: A rare entity with a complex diagnosis. Rev Port Cardiol. 2023;42(2):169.e1-169.e4. English, Portuguese. 10.1016/j.repc.2022.12.007 36526128

[13] Lee MH, Lee HB, Lee YC, Rhee YK, Lee EJ, Chung MJ, Jin GY, Kweon EY, Park SJ (2011). Bilateral multiple inflammatory myofibroblastic tumors of the lung successfully treated with corticosteroids. Lung.

[14] Andersen ND, DiBernardo LR, Linardic CM, Camitta MG, Lodge AJ (2012). Recurrent inflammatory myofibroblastic tumor of the heart. Circulation.

[15] Karnak I, Senocak ME, Ciftci AO, Cağlar M, Bingöl-Koloğlu M, Tanyel FC, Büyükpamukçu N (2001). Inflammatory myofibroblastic tumor in children: diagnosis and treatment. J Pediatr Surg.

[16] Baldi GG, Brahmi M, Lo Vullo S, Cojocaru E, Mir O, Casanova M, Vincenzi B, De Pas TM, Grignani G, Pantaleo MA, Blay JY, Jones RL, Le Cesne A, Frezza AM, Gronchi A, Collini P, Dei Tos AP, Morosi C, Mariani L, Casali PG, Stacchiotti S (2020). The activity of Chemotherapy in Inflammatory Myofibroblastic tumors: a Multicenter, European Retrospective Case Series Analysis. Oncologist.

[17] Coffin CM, Hornick JL, Fletcher CD (2007). Inflammatory myofibroblastic tumor: comparison of clinicopathologic, histologic, and immunohistochemical features including ALK expression in atypical and aggressive cases. Am J Surg Pathol.

[18] Schöffski P, Sufliarsky J, Gelderblom H, Blay JY, Strauss SJ, Stacchiotti S, Rutkowski P, Lindner LH, Leahy MG, Italiano A, Isambert N, Debiec-Rychter M, Sciot R, Van Cann T, Marréaud S, Nzokirantevye A, Collette S, Wozniak A (2018). Crizotinib in patients with advanced, inoperable inflammatory myofibroblastic tumours with and without anaplastic lymphoma kinase gene alterations (European Organisation for Research and Treatment of Cancer 90101 CREATE): a multicentre, single-drug, prospective, non-randomised phase 2 trial. Lancet Respir Med.

[19] Mahajan P, Casanova M, Ferrari A, Fordham A, Trahair T, Venkatramani R (2021). Inflammatory myofibroblastic tumor: molecular landscape, targeted therapeutics, and remaining challenges. Curr Probl Cancer.

